# Using red blood cell distribution width to predict death after abdominal aortic aneurysm rupture

**DOI:** 10.1186/s12872-023-03191-1

**Published:** 2023-03-30

**Authors:** Wanghai Li, Tao Liao, Yan Zhang, Chengzhi Li

**Affiliations:** 1 Department of Interventional Radiology and Vascular Surgery, The First Affiliated Hospital of Jinan University, Guangzhou, 510630 China; 2 Department of Critical Care Medicine, Renmin Hospital, Hubei University of Medicine, Shiyan City, 442000 People’s Republic of China

**Keywords:** Rupturned abdominal aortic aneurysm, All-cause mortality, Red blood cell distribution, MIMIC-III, Predictive models

## Abstract

**Background:**

An abdominal aortic aneurysm is a life-threatening enlargement in the major vessel at the abdomen level. This study investigated the associations between different levels of red blood cell distribution width and all-cause mortality among patients with abdominal aortic aneurysm rupture. It developed predictive models for all-cause mortality risk.

**Methods:**

This was a retrospective cohort study using 2001 to 2012 MIMIC-III dataset. The study sample included 392 U.S. adults with abdominal aortic aneurysms who were admitted to ICU after the aneurysm rupture. Then we used two single-factor and four multivariable logistic regression models to examine the associations between different levels of red blood cell distribution and all-cause mortality (30 days and 90 days), controlling for demographics, comorbidities, vital signs, and other laboratory measurements. The receiver operator characteristic curves were calculated, and the areas under the curves were recorded.

**Results:**

There were 140 (35.7%) patients with an abdominal aortic aneurysm in the red blood cell distribution width range between 11.7 and 13.8%, 117 (29.8%) patients in the range between 13.9 and 14.9%, and 135 (34.5%) patients in the range between 15.0 and 21.6%. Patients with higher red blood cell distribution width level (> 13.8%) tended to have a higher mortality rate (both 30 days and 90 days), congestive heart failure, renal failure, coagulation disorders, lower hemoglobin, hematocrit, MCV, red blood cell count, higher levels of chloride, creatinine, sodium, and BUN (All P < 0.05). Results of multivariate logistic regression models indicated that patients with higher red blood cell distribution width levels (> 13.8%) had the highest statistically significant odd ratios of 30 days and 90 days of all-cause mortality than lower red blood cell distribution width levels. The area under the RDW curve was lower (*P* = 0.0009) than that of SAPSII scores.

**Conclusions:**

Our study found that patients with abdominal aortic aneurysm rupture with a higher blood cell distribution had the highest risk of all-cause mortality. Using the blood cell distribution width level in patients with abdominal aortic aneurysm rupture to predict mortality should be considered in future clinical practice.

## Background

An abdominal aortic aneurysm (AAA) is a life-threatening enlargement in the major vessel at the abdomen level. Previous studies indicated that the prevalence of AAA in men older than 60 ranged from 1.2 to 3.3% [1–3]. Because most AAAs are asymptomatic, the associated risk for death is up to 81% when they rupture. Therefore, there have been widespread efforts to develop safe and effective screening methods to detect AAA at the curable stage to reduce its mortality. The 2019 US Preventive Services Task Force recommends 1-time screening for AAA in individuals aged 65–75 years who ever smoked [4]. An estimated risk of AAA mortality could be reduced by up to 35% in the USA if 1-time screening with ultrasonography is widely used in the eligible population [1]. However, data has shown pervasive underutilization of the recommended 1-time screening with ultrasonography [5]. It suggests that many high-risk individuals do not receive qualified screening. Previous studies indicated there are multiple barriers to implementing the ultrasonography screen. Two barriers are the availability of the screening test and the high cost. Therefore, more cost-effective risk assessment methods are urgently needed to identify high-risk individuals and optimize the AAAs screening criteria.

The wide use of Electronic Healthcare Records (EHRs) has provided new opportunities for population-based studies on a large-scale dataset. Typical EHR systems contain various data sources, including claim data, laboratory and imaging data, medication records, and clinical diagnoses. The rise of widely used EHRs provides promising opportunities for applying existing risk evaluation models and developing and refining new risk prediction models [6, 7]. The advantages of using EHRs to create a risk prediction model include: (1) EHR data include large numbers of patients, allowing one to create more comprehensive and accurate models based on the large metrics; (2) EHRs-based populations may reflect the real-world situation more than cohort studies that rely on volunteer participation and selection bias; (3) prediction models based on EHRs data may be implemented directly rather than first translated to a clinical environment in those traditional models; (4) implementing real-world-based risk prediction models in the EHRs significantly increases the possible clinical utility of those models by making them immediately available during health care and reducing the need for clinicians to calculate the risk manually. Using an accurate model that incorporates additional factors, such as biological markers, may identify more persons who have AAA or in whom AAA may rupture, possibly reducing the risk of mortality. Red blood cell distribution width (RDW), as a part of the standard complete blood count test, is used to measure the range of variation of erythrocytes. Recent evidence shows that RDW may be potentially used as a predictor of various chronic diseases [8–10], or to predict the outcomes of pulmonary hypertension [8], heart failure [9], acute kidney injury (AKI) [10], tumors [11], and others [12, 13]. Because of the low cost of a complete blood count test and the association with cardiovascular diseases, the RDW may be used as an alternative mortality prediction method in patients with AAA. However, no study has examined the predictive effects of RDW on mortality among AAA patients with rupture. This study investigated the associations between different levels of RDW and all-cause mortality among patients with AAA rupture. Then we developed predictive models for AAA rupture data analytics and discovery, focusing on refining and developing all-cause mortality risk prediction models using nationally representative dataset.

## Methods

### Study sample

This was a retrospective cohort study design using the 2001 to 2012 Multiparameter Intelligent Monitoring in Intensive Care Database III version 1.3 (MIMIC-III v1.3) dataset, which is a large-scale HER data of more than 50,000 intensive care unit (ICU) patients at Beth Israel Deaconess Medical Center (Boston, MA, USA). The MIMIC-III dataset includes information on International Classification of Diseases (ICD-9) codes, medications (generic drug name and NDC codes), Current Procedural Terminology codes, laboratory and imaging reports, vital signs, the length of hospitalizations, and death date. All MIMIC-III users had to finish the National Institutes of Health Protecting Human Research Participants training course and pass the qualified test to register for the database. After completing the above requirements, we obtained approval from the MIMIC-III database administration staff (Certification Number: 39,692,708).

For this study, we used the PATIENTS file and ADMISSIONS file in the MIMIC-III dataset to identify the participants. DIAGNOSES_ICD, CPTEVENTS, PROCEDURES_ICD, CHARTEVENTS, and PRESCRIPTIONS files are core files collecting information on diagnosis, procedures, all charted observations among respondents, and medications ordered for a given patient. The laboratory measurements for patients, microbiology culture results, ECG reports, and radiology reports are included in the files of LABEVENTS, MICROBIOLOGYEVENTS, and NOTEEVENTS. Furthermore, the information about the ICU stay is contained in the ICUSTAYS file. A detailed description of the MIMIC-III samples design and the data collection is available from the MIMIC-III dataset description documents.

We used the PostgreSQL tool (version 9.6) to extract data from the MIMIC-III dataset. The data information we pulled included the information from clinical, laboratory, demographic, and scoring systems. Specifically, the clinical information was used to identify the comorbidities, which included chronic obstructive pulmonary disease, congestive heart failure, coronary artery disease, atrial fibrillation, stroke, AKI, pneumonia, and liver disease. Laboratory measurements included creatinine, blood urea nitrogen (BUN), platelets, prothrombin time (PT), activated partial thromboplastin time, international normalized ratio (INR), white blood cells, hemoglobin, hematocrit, bicarbonate, sodium, chloride, potassium, anion gap, chloride, and glucose. The scoring systems included sequential organ failure assessment (SOFA) and the simplified acute physiology score II (SAPS II), which were usually used to predict the progress of ICU patients’ outcomes. All the above data were extracted within 24 h after ICU admission. Social Security Death Index was used to identify the 30-day and 90-day mortality.

The study population was limited to U.S. adults (aged ≥ 18) with concurrent AAA rupture. All participants were classified as having current AAA rupture using the ICD-9 code (ICD-9: 441.3 and 441.4). If patients did not have RDW measurements during ICU stay or the missing values > 5% in the individual data, they were excluded. The final study sample size included 392 adults. This study was approved by the institutional review board of the First Affiliated Hospital of Jinan University.

### Measurements

The RDW was measured as a continuous variable after AAA rupture. The mean of RDW results within 24 h after ICU admission was further categorized in mutually exclusive categories: (1) 11.7-13.8%; (2) 13.9-14.9%; (3) 15.0-21.6%.

### Main outcome measures

The primary outcomes of interest were clinical outcomes of patients with AAA rupture, including all-cause 30 days and 90 days mortality. All-cause mortality was identified using the date of death available in the MIMIC-III dataset.

### Covariates

The covariates of patients with rAAA included baseline demographics (age and race), the length of ICU stay, other laboratory measurements, residence in an urban or rural area, socioeconomic status, tumor characteristics, and comorbid conditions. We also collected the vital signs of the first 24 h of ICU stay, including heart rate, systolic blood pressure (SBP), mean arterial pressure, temperature, respiratory rate, and oxyhemoglobin saturation (SpO^2^). The Sequential organ failure assessment (SOFA) and the SAPS II were also collected. Race (white, black, and others) was categorized. Other laboratory measurements were collected as continuous variables, which included hemoglobin, glucose, anion gap, bicarbonate, chloride, creatinine, potassium, sodium, BUN, hematocrit, INR, PT, MCV, platelet, and red blood cells. The baseline comorbidities were collected using specific ICD-9-CM codes one month before rAAA diagnosis.

### Statistical analyses

Baseline patient characteristics were compared using the Chi-square test for categorical variables and t-test for continuous variables across patients with three different levels of RDW.

Then we conducted two signal variable and four multivariable logistic regression models to test the associations between different RDW levels and 30 and 90 days mortality among adult rAAA patients (model 1, 2, and 3–0 days mortality; model 4, 5, and 6–90 days mortality). Models 1 and 4 were single variables. The multivariable models 2 and 5 were adjusted for covariates and included sex, race, and age. The multivariable models 3 and 6 were adjusted for covariates and included sex, race, age, temperature, SPO2, glucose, hemoglobin, anion gap, bicarbonate, creatinine, SOFA, SAPSII, and comorbidities.

The receiver operator characteristic (ROC) curves of the adjusted odds ratio (AOR) of 30 and 90 days of all-cause mortality were calculated. The area under the curve (AUC) and the 95% confidence interval were recorded. Delong’s method was used to test whether the difference between the ROC curves was statistically significant.

We also performed two sensitivity analyses to examine our findings’ robustness by differing across various subgroups, including gender, ethnicity, congestive heart failure, hypertension, renal failure, coagulopathy, hemorrhagic anemia, and renal replacement therapy. All analyses used SPSS 23.0 with *P* < 0.05 as statistical significance.

## Results

Among the final cohort (n = 392), there were 140 (35.7%) patients with rAAA in the RDW range between 11.7 and 13.8%, 117 (29.8%) rAAA patients in the RDW range between 13.9 and 14.9%, and 135 (34.5%) rAAA patients in the RDW range between 15.0 and 21.6% (Table 1). The mean age among three groups of rAAA patients was 72.56 (SD = ± 22.37), 76.28 (SD = ± 9.25), and 75.95(SD = ± 9.27), respectively. Patients with higher RDW levels (> 13.8%) tended to have a higher mortality rate (both 30 and 90 days), congestive heart failure, renal failure, coagulation disorders, lower hemoglobin, hematocrit, MCV, red blood cell count, higher level of chloride, creatinine, sodium, and BUN (All *P* < 0.05). In addition, the mean scores of SOFA and SAPSII, and 30 and 90-day mortality were also statistically significant among three groups of rAAA patients. Specifically, compared with the other two groups of rAAA patients, the patients in the RDW range between 11.7 and 13.8% had lower scores of SOFA (5.04, SD = ± 3.13 vs. 5.74, SD = ± 2.81, vs. 6.46, SD = ± 3.14) and SAPSII (37.84, SD = ± 13.27 vs. 41.19, SD = ± 14.51 vs. 44.07, SD = ± 15.13), and 30-days (8.57% vs. 17.09%, vs. 20.74%) and 90-days (11.43% vs. 23.93%, vs. 25.93%) mortality (All *P* < 0.05).

**Table 1:** Characteristics of the study patients according to RDW

Characteristics	11.7–13.8	RDW, % 13.9–14.9	15.0-21.6	*P* value
Clinical parameters, n	140	117	135	
Age, years	72.56 ± 22.37	76.28 ± 9.25	75.95 ± 9.27	0.089
Gender, n (%)				0.252
Female	42(30.00)	43(36.75)	53(39.26)	
Male	98(70.00)	74(63.25)	82(60.74)	
Ethnicity, n (%)				0.8552
White	111(79.29)	95(81.20)	110(81.48)	
Black	5(3.57)	2(1.70)	5(3.70)	
Other	24(17.14)	20(17.09)	20(14.81)	
ICU stay, days	6.49 ± 9.40	6.23 ± 7.04	6.55 ± 8.36	0.951
SBP, mmHg	117.46 ± 14.00	118.46 ± 14.18	117.91 ± 15.42	0.407
MBP, mmHg	78.41 ± 8.80	77.47 ± 8.39	77.63 ± 9.84	0.659
Heart rate, beats/minute	80.58 ± 11.94	83.52 ± 13.54	81.94 ± 15.36	0.229
Respiratory rate, beats/minute	17.48 ± 3.14	18.27 ± 3.10	17.76 ± 3.31	0.136
Temperature, ∘C	36.87 ± 0.71	36.86 ± 0.66	36.70 ± 0.68	0.071
SPO2, %	97.13 ± 1.97	97.29 ± 1.87	97.38 ± 1.89	0.564
Comorbidities, n (%)				
Congestive heart failure	34(24.29)	37(31.62)	61(45.19)	**0.001**
Hypertension	98(70.00)	84(71.79)	97(71.85)	0.929
Renal failure	5(3.57)	18(15.38)	36(26.67)	**< 0.001**
Coagulopathy	17(12.14)	15(12.82)	38(28.15)	**< 0.001**
Hemorrhagic anemia	4(2.86)	2(1.71)	8(5.93)	0.169
Renal replacement therapy	0(0)	3(2.56)	4(2.96)	0.134
Laboratory parameters				
Hemoglobin,g/dl	12.05 ± 2.09	11.28 ± 1.98	10.83 ± 2.21	**< 0.001**
Glucose, mg/dl	143.48 ± 66.76	142.82 ± 55.78	138.91 ± 53.81	0.792
Anion gap, mmol/l	14.17 ± 3.86	13.88 ± 4.13	14.53 ± 4.12	0.437
Bicarbonate, mmol/L	24.13 ± 3.90	23.90 ± 4.88	23.10 ± 4.91	0.154
Chloride, mmol/L	104.92 ± 5.45	105.29 ± 6.98	106.89 ± 6.72	**0.027**
Creatinine,mEq/L	1.05 ± 0.41	1.17 ± 0.58	1.42 ± 0.95	**< 0.001**
Potassium, mmol/L	4.25 ± 0.67	4.37 ± 0.75	4.35 ± 0.74	0.309
Sodium, mmol/L	138.91 ± 3.58	138.52 ± 4.18	140.11 ± 4.16	**0.004**
BUN, mg/dl	19.29 ± 8.80	22.42 ± 12.04	27.07 ± 15.70	**< 0.001**
Hematocrit, %	35.86 ± 5.50	34.11 ± 5.87	32.69 ± 6.52	**< 0.001**
INR	1.41 ± 0.68	1.56 ± 1.63	1.54 ± 0.81	0.451
PT, second	14.89 ± 4.71	16.21 ± 12.16	16.17 ± 6.22	0.314
MCV, fL	91.25 ± 5.55	89.94 ± 4.46	89.32 ± 7.36	**0.024**
Platelet, 10^9^/L	212.33 ± 78.44	188.05 ± 78.99	209.99 ± 123.87	0.108
Red Blood Cells, 10^12^/L	3.94 ± 0.63	3.79 ± 0.69	3.69 ± 0.81	0.018
Scoring systems				
SOFA	5.04 ± 3.13	5.74 ± 2.81	6.46 ± 3.14	**0.001**
SAPSII	37.84 ± 13.27	41.19 ± 14.51	44.07 ± 15.13	**0.002**
30-day mortality, n (%)	12(8.57)	20(17.09)	28(20.74)	**0.016**
90-day mortality, n (%)	16(11.43)	28(23.93)	35(25.93)	**0.005**

### Adjusted associations between RDW levels and mortality

Results of multivariate logistic regression models for adjusted associations between patients’ RDW levels and all-cause mortality are presented in Table 2. Overall, patients with higher RDW level (> 13.8%) had the highest statistically significant odd ratios of 30 days all-cause mortality (OR = 2.51, 95% CI = 1.28, 4.91; AOR = 2.54, 95% CI = 1.28, 5.04; AOR = 2.25, 95% CI = 1.05, 4.81) and 90 days all-cause mortality (OR = 2.58, 95% CI = 1.43, 4.68; AOR = 2.50, 95% CI = 1.34, 4.66; AOR = 2.22, 95% CI = 1.12, 4.41) compared with lower RDW level.

**Table 2:** Associations between the RDW and all-cause mortality (30 days and 90 days)

RDW, %	Non-adjusted		Model I		Model II	
	OR (95%CIs)	P value	OR (95%CIs)	P value	OR (95%CIs)	P value
30-day mortality						
Fitted group						
< 13.85	1.0(ref)		1.0(ref)		1.0(ref)	
≥ 13.85	2.51(1.28,4.91)	0.0057	2.54(1.28,5.04)	0.008	2.25(1.05,4.81)	0.037
90-day mortality Fitted group						
< 13.85	1.0(ref)		1.0(ref)		1.0(ref)	
≥ 13.85	2.58(1.43,4.68)	0.0013	2.50(1.34,4.66)	0.004	2.22(1.12,4.41)	0.022

### Prediction of mortality using RDW

**Fig. 1:**
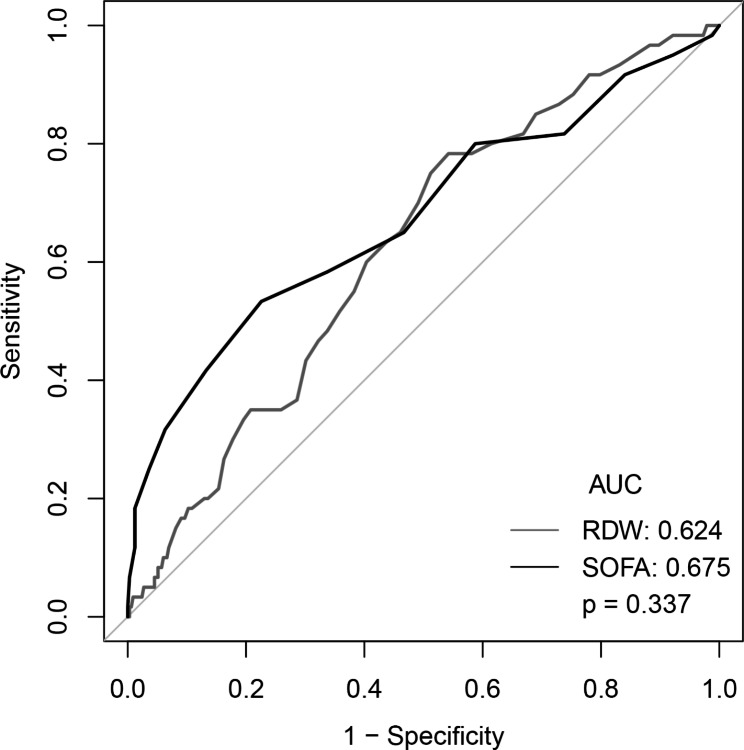
ROC curve for logistic regression model and SOFA score

**Fig. 2:**
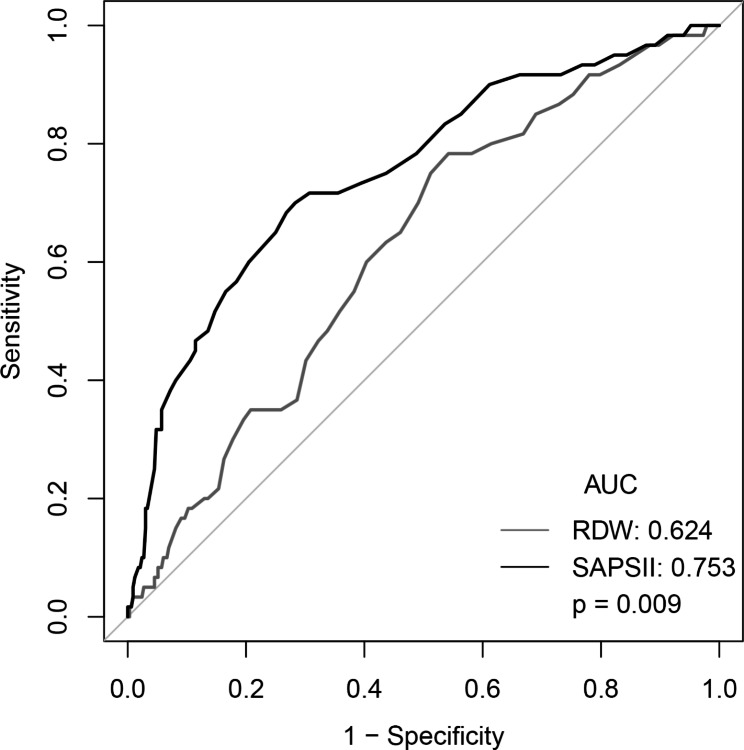
ROC curve for logistic regression model and SOFA score

We calculated the AUC of various indicated variables (RDW, SOFA scores, and SAPSII scores) among AAA patients to predict 30 and 90 days all-cause mortality (Figs. 1 and 2). The AUCs for RDW and SOFA scores and SAPSII scores were 0.624, 0.675, and 0.753, respectively. Compared to the AUC of SAPSII scores, the AUC of the RDW was lower (*P* = 0.0009). However, there was no statistical significance between the AUC of the RDW and the AUC of SOFA scores (*P* = 0.337).

Two sets of sensitivity analyses were performed to compare with the main results, and both found similar results. Specifically, we included gender, ethnicity, congestive heart failure, hypertension, renal failure, coagulopathy, hemorrhagic anemia, and renal replacement therapy for subgroup analysis. The association between RDW and the risk of 30-day mortality was different for these strata. RDW showed no significant interactions with gender (*P* = 0.097), hypertension (*P* = 0.277), and hemorrhagic anemia (*P* = 0.775) for 30-day mortality. However, RDW had significant interactions with ethnicity (*P* = 0.003), congestive heart failure (*P* = 0.001), renal failure (*P* = 0.033), coagulopathy (*P* = 0.003), and renal replacement therapy (*P* = 0.001) for 30-day mortality.

The association between RDW and the risk of 30-day mortality was similar for the white population and patients without congestive heart failure, renal failure, and renal replacement therapy. The higher RDW was significantly associated with the higher 30-day mortality in the white population (AOR = 3.165, 95% CI = 1.359–7.373), patients without congestive heart failure (AOR = 4.708, 95% CI = 1.583–13.999), patients without renal failure (AOR = 2.125, 95% CI = 1.057–4.272), and patients without renal replacement therapy (AOR = 2.207, 95% CI = 1.120–4.350). However, there was no significant relationship between RDW and 30-day mortality in the black and other populations, patients with congestive heart failure, and patients with renal failure (*P* > 0.05).

## Discussion

In this large population-based study, we observed the adverse effects of higher (> 13.8%) red blood cell distribution width (RDW) level on mortality (both 30 and 90 days) among patients with ruptured abdominal aortic aneurysm (rAAA). These effects remained after controlling for patient’s characteristics and vital signs, other laboratory measurements, and comorbidities. Although the AUC of the RDW was lower than SOFA and SAPSII scores, it had a certain predictive performance.

This study found that patients with higher RDW levels tend to have more comorbidities, such as congestive heart failure, renal failure, and coagulation disorders. We also observed that they had more abnormal laboratory results than patients with lower RDW levels. Specifically, they were more likely to have lower hemoglobin and hematocrit, higher levels of MCV, lower red blood cell count, and a higher level of chloride, creatinine, sodium, and BUN. These findings were consistent with previous studies. Our findings supported that the overall health status in rAAA patients with higher levels of RDW was worse compared with patients in the reference group, and multiple chronic conditions have been associated with an increased risk of mortality. Indeed, compared with those rAAA patients without multiple chronic conditions who were admitted into ICU, those with multiple conditions might stay in ICU longer. In addition, rAAA patients with multiple chronic conditions may not tolerate AAA repair surgeries [14]. For example, based on a study from Aggarwal and colleagues, approximately 1,400 AAA patients die due to AAA repair surgeries annually in the U.S. to prevent rupture [15–17]. Previous studies indicated that medications for hypertension and hyperlipidemia might reduce AAA growth [18, 19]. Although the mechanism is unknown, several theories may partially explain it, including lowering the intramural aortic MMP expression and degree of inflammation [18]. Moreover, patients with multiple conditions may be related to medication adherence issues. Lower medication adherence with those multiple chronic conditions may make those conditions worse.

We also observed that our study’s 30- and 90-day mortality rates were higher than the general population. For example, previous studies found that the range of perioperative mortality was 1–5% [20–24]. We also observed lower RDW levels associated with a lower mortality rate for long-term outcomes. Our findings suggest that mortality is related to elevated RDW levels among rAAA patients admitted into ICU compared with RDW in the general population. This discrepancy between different levels of RDW and all-cause mortality in our study needs to be thoroughly investigated. Future studies may consider exploring the factors that may affect the progress of AAA and explain this finding.

Although AAA is a rare disease in younger adults, among older males (> 65 years old), the prevalence of AAA is up to 8% [25–27]. Because most AAA are asymptomatic before the rupture, AAA patients who had the rupture are potentially lethal. Therefore, based on a population estimate, each year, 4,500 AAA patients die due to the rupture, and it is the 14th leading cause of mortality in the United States [15]. The risk factors associated with AAA include older age, male, smoking history, family history of AAA, cardiovascular conditions, and risk factors such as coronary artery disease, cerebrovascular disease, atherosclerosis, hypercholesterolemia, and hypertension [28–31]. A higher level of RDW, as an independent risk factor, has been indicated to be associated with multiple cardiovascular conditions and risk factors by many studies [8, 10, 11, 32, 33]. Our study extended this research and showed an association between a high level of RDW and mortality risk among patients with rAAA. One possible explanation for the increased risk of all-cause mortality among rAAA patients with a higher level of RDW is that a higher level of RDW may indirectly reflect organ dysfunctions. Indeed, the RDW is associated with systemic inflammation, which is common among patients with multiple organ dysfunctions. Previous studies found that systemic inflammation would inhibit erythrocyte maturation. In addition, oxidative stress, which is common among patients with organ dysfunction, will decrease the survival rate of erythrocytes and release premature erythrocytes into circulation. All the above pathological mechanics may relatively cause an increase in RDW.

Our sensitivity analyses found that sex differences in mortality might exist. Our study found that the 30-day mortality rate in men was statistically more significant than in women. However, the interaction between men and women was not significant. These findings were inconsistent with previous studies [34] which indicated that women were more likely to die than men due to the AAA rupture. One reason is that women are less likely to be admitted to the hospital and receive qualified management [35]. In our study, we found that if women were admitted to the hospital, the mortality rate might not differ from men. However, we only analyzed 30-day mortality; the 90-day mortality rate may still exist due to the sex difference. Therefore, future studies may investigate the sex difference in mortality in AAA patients with rupture.

In this study, we also developed a predictive model to predict future all-cause mortality of rAAA. We used the well-validated SOFA and SAPSII scores as a baseline for the performance comparison and the AUC as an overall measure of discrimination. Our result is promising: This model using RDW is more accurate (AUC: xxx) for early detection of all-cause mortality than either SOFA scores for screening or the SAPSII scores. A similar result has been reported recently among other studies of all-cause mortality. However, we did not observe a significant difference between RDW and SOFA scores for predicting the risk of all-cause mortality among patients with rAAA. This finding contrasts with previous studies that suggested that RDW was a better predictor than SOFA scores for predicting the risk of worse clinical outcomes, such as mortality. In addition, SOFA and SAPSII scores predict mortality for all patients admitted to the ICU. Our rAAA mortality prediction models are developed only for ICU patients with rAAA and are based on different clinical variables. Therefore, our rAAA preoperative models can predict up to 90 days mortality, which is not available for ICU scores. However, there are complex associations between RDW and mortality, and much remains unknown. Therefore, studies examining the predictive performance for all-cause mortality using RDW after AAA rupture are needed to be investigated in more studies.

Finally, based on our finding, RDW could be used in real clinical practice for screening and risk stratification. Specifically, patients with AAA could be screened for elevated RDW levels as part of their routine blood work. This could help identify patients at higher risk of mortality and guide appropriate management strategies. In addition, patients with ruptured AAA could be stratified based on their RDW levels to help determine their risk of mortality. Finally, there are various factors that can affect RDW, including vitamin B12 deficiency, iron deficiency, and other nutritional deficiencies. As part of the modifiable risk factor, the underlying cause of anemia or blood disorder must be identified and addressed. For example, Vitamin B12 or iron deficiency can be treated with supplements or dietary changes. This could help clinicians decide on appropriate treatment strategies, including surgical intervention and postoperative care.

### Limitations

There are several limitations and strengths in our study. First, selection bias or unmeasurable confounding factors may exist in the observational study. For example, we could not measure other factors potentially associated with mortality, such as medications use, imaging reports, or genetic biomarkers that might affect a provider’s treatment decision-making. Second, the nature of registry and claims data might introduce biases, such as ascertainment of clinical outcomes. However, diagnosis codes and various laboratory measurements in MIMIC-III datasets are widely accepted in previous studies focused on patients with various diseases. Third, there may be potential confounders of RDW measurements after rAAA diagnosis, with various durations. However, we addressed this issue by conducting sensitivity analyses limiting to patients within ICU admission periods. Similar to the main results, patients with a high level of RDW still had the highest statistically significant risks of worse clinical outcomes. Finally, our study sample was limited to single-center areas; therefore, results may not be generalized to other populations not included in this study.

## Conclusions

In conclusion, our study found that patients with AAA rupture with a higher level of RDW had the highest risks of worse clinical outcomes. Future clinical practice should consider the decision to use the RDW level in patients with rAAA to predict mortality.

## Data Availability

The datasets used and/or analysed during the current study are available from the corresponding author on reasonable request.

## Abbreviations

AAA: Abdominal aortic aneurysm
EHRs: Electronic Healthcare Records
RDW: Red blood cell distribution width
AKI: Kidney injury
ICU: Intensive care unit
ICD-9: International Classification of Diseases
MIMIC-III: Multiparameter Intelligent Monitoring in Intensive Care Database III
BUN: Blood urea nitrogen
PT: Prothrombin time
INR: International normalized ratio
SAPS II: Simplified acute physiology score II
SBP: Systolic blood pressure
SpO^2^: Oxyhemoglobin saturation
SOFA: Sequential organ failure assessment
ROC: Receiver operator characteristic
AOR: Adjusted odds ratio
AUC: Area under the curve
rAAA: Ruptured abdominal aortic aneurysm
